# Isolation and Characterization of Eleven Polymorphic Microsatellite Loci for the Valuable Medicinal Plant *Dendrobium huoshanense* and Cross-Species Amplification

**DOI:** 10.3390/ijms131216779

**Published:** 2012-12-07

**Authors:** Hui Wang, Nai-Fu Chen, Ji-Yang Zheng, Wen-Cai Wang, Yun-Yun Pei, Guo-Ping Zhu

**Affiliations:** 1 Key Laboratory of Molecular Evolution and Biodiversity and Anhui Provincial Key Laboratory of the Conservation and Exploitation of Biological Resources, College of Life Science, Anhui Normal University, Wuhu 241000, China; E-Mails: wh741230@163.com (H.W.); jiyangaq@163.com (J.-Y.Z.); wangwencai19872006@126.com (W.-C.W.); peiyy923@126.com (Y.-Y.P.); 2 Engineering Technology Research Center of Plant Cell Engineering of Anhui Province, Lu’an 237012, China; E-Mail: chennf@163.com

**Keywords:** *Dendrobium huoshanense*, medicinal plant, microsatellite, molecular marker, cross-amplification, population genetics

## Abstract

*Dendrobium huoshanense* (Orchidaceae) is a perennial herb and a widely used medicinal plant in Traditional Chinese medicine (TCM) endemic to Huoshan County town in Anhui province in Southeast China. A microsatellite-enriched genomic DNA library of *D. huoshanense* was developed and screened to identify marker loci. Eleven polymorphic loci were isolated and analyzed by screening 25 individuals collected from a natural population. The number of alleles per locus ranged from 2 to 5. The observed and expected heterozygosities ranged from 0.227 to 0.818 and from 0.317 to 0.757, respectively. Two loci showed significant deviations from Hardy-Weinberg equilibrium and four of the pairwise comparisons of loci revealed linkage disequilibrium (*p* < 0.05). These microsatellite loci were cross-amplified for five congeneric species and seven loci can be amplified in all species. These simple sequence repeats (SSR) markers are useful in genetic studies of *D. huoshanense* and other related species and in conservation decision-making.

## 1. Introduction

*Dendrobium huoshanense*, a perennial epiphytic orchid herb, is endemic to Huoshan County town in Anhui province in southeast China. It is a widely used medicinal plant in traditional Chinese medicine (TCM), whose stems have been used for centuries in China and Southeast Asia as a therapeutic agent for treatment of cataract, throat inflammation, fever, chronic superficial gastritis, and as a tonic to improve health, mentality and to delay aging [1]. Recent researches revealed that the pharmacological activity of *D. huoshanense* was due to several active components, such as alkaloids, stilbenoid, glycosides and polysaccharides [2–4]. Moreover, *D. huoshanense* is of great horticultural interest due to the delicate lime flowers that bloom from May [4]. However, human activities have brought this species to the brink of extinction in the last few decades. Extensive deforestation and increased utilization in TCM have caused a dramatic decline in the natural populations. Although the morphology and pharmacology of *D. huoshanense* has been extensively studied, information on molecular phylogeny and population genetics is largely unknown.

During recent decades, SSRs (simple sequence repeats), also known as microsatellites, have become the most popular source of genetic markers owing to their high reproducibility, multi-allelic nature, codominant inheritance, high abundance, and wide genome coverage [5,6]. They are an ideal tool for genome mapping, population genetics, conservation biology, marker-assisted selection and other studies [7–10]. Previous work by Lu *et al.* showed the development and characterization of SSR markers for *Dendrobium officinale*[11]. In addition, Ding *et al.* reported that 13 of the 15 tested *D. officinale* SSR primer pairs were successful in amplifying corresponding products in *D. huoshanense*[12]. Here, we report the isolation and characterization of eleven polymorphic microsatellite loci from *D. huoshanense* using a modified biotin-capture method for the first time [13].

## 2. Results and Discussion

Eleven polymorphic microsatellite loci were isolated and deposited in GenBank (JQ400190–JQ400200; Table 1). Polymorphism at each locus was determined using 25 *D. huoshanense* individuals from a natural population. The number of alleles per locus ranged from two to five. The largest number of alleles (five) was found at Hs48 locus (Table 1). The observed heterozygosity (*H*_o_) ranged from 0.227 to 0.818 and the expected heterozygosity (*H*_e_) ranged from 0.317 to 0.757 (Table 1).

Deviations from Hardy-Weinberg expectation (HWE) were detected for each locus, nine loci conformed to HWE and two loci (Hs39, Hs50) significantly deviated from HWE in the sampled population after Bonferroni’s correction (*p* < 0.05). Linkage disequilibrium (LD) is important for evolutionary and population genetics and so LD between pairs of loci was calculated. Four of the pairwise comparisons (Hs40 and Hs47, Hs41 and Hs54, Hs50 and Hs57, Hs48 and Hs54) exhibited significant linkage disequilibrium (*p* < 0.05).

The cross-species amplification was carried out. The results of the screen are summarized in Table 2. Notably, seven loci (Hs39, Hs41, Hs44, Hs47, Hs48, Hs56, Hs57) can be amplified in all five species, while three loci (Hs40, Hs50, Hs54) can be amplified in four species and one loci (Hs42) can be amplified in two species. These results suggest that the identified microsatellite markers are useful in genetic studies of *D. huoshanense* and other *Dendrobium* species.

## 3. Experimental Section

### 3.1. Plant Materials and DNA Extraction

The leaves of *D. huoshanense* from a natural population in Huoshan (31°0′35″N, 115°0′53″E, Anhui, China) were collected, dried in silica gel in sealed polyethylene bags and stored at room temperature until use. The total genomic DNA of 25 individuals was extracted using the standard cetyltrimethyl ammonium bromide (CTAB) method [14].

### 3.2. Isolation of Microsatellite Markers

About 500 ng genomic DNA from a single individual were digested by *Sau*3AI (TaKaRa), and the fragments ranging from 400 to 900 bp were purified and ligated to the *Sau*3AI adaptors oligoA (5′-GGCCAGAGACCCCAAGCTTCG-3′) and oligoB (5′-PO4-GATCCGAAGCTTGGGGTCTCTGG CC-3′) [13]. The products were then hybridized to the single-stranded 3′-biotinylated (CT)_15_ oligonucleotide probes and captured by streptavidin-coated beads (Promega). Captured fragments were amplified and ligated to pMD18-T vector (TaKaRa). The recombinant plasmid was transformed into *Escherichia coli* DH5α and the transformants were distinguished by blue-white screening. The insert-positive clones were identified by PCR using oligo(A) and (CT)_15_ oligonucleotides as the primers. The clones that yielded two or more bands contained microsatellite fractions.

In total, fifty-one positive colonies were sequenced on the ABI-PRISM 3730 automated sequencer, and thirty-six contained microsatellites. Thirty-six pairs of primers were then designed using PRIMER PREMIER 5.0 software [15] and synthesized. Eleven sets of primers that gave consistent and specific PCR products were tested for allelic polymorphism in 25 individuals from a natural population. PCR amplifications (15 μL) contained 6.25 μL GoTaq^®^ Green Master Mix (Promega, Nanjing, China), 0.5–1.0 μmol of each primer and 10–20 ng DNA, and were performed in an iCycler thermocycler (Bio-Rad). PCR conditions were 4 min at 94 °C followed by 35 cycles of 45 s at 94 °C, 35 s at optimized annealing temperature (Table 1) and 30 s at 72 °C, with a final extension time of 8 min at 72 °C. PCR products were separated on an 8% denaturing polyacrylamide gel using a LI-COR 4200 automated DNA sequencer and analyzed using LI-COR SAGA^GT^ software.

### 3.3. Data Analysis

The number of alleles (*N*_a_) per locus, observed heterozygosity (*H*_o_) and expected heterozygosity (*H*_e_), Hardy-Weinberg equilibrium (HWE) and linkage disequilibrium (LD) were tested using GENEPOP version 4.0 [16]. MICRO-CHECKER version 2.2.3 was used to determine the presence of null alleles [17]. All results for multiple tests were corrected using Bonferroni’s correction [18].

### 3.4. Cross-Species Transferability

Cross-species amplification of microsatellite markers was performed for five congeneric species, *D. aduncum*, *D. henanense*, *D. moniliforme*, *D. nobile and D. officinale*. DNA extraction and PCR amplification were performed as described for *D. huoshanense* except that the annealing temperature was re-optimized for each locus. Five individuals from each species were screened at 11 microsatellite loci.

## 4. Conclusions

We report the isolation and characterization of eleven polymorphic microsatellite loci for *D. huoshanense* and cross-amplification in five congeneric species. The low level of polymorphism here was possibly caused by heterozygote deficiency resulting from inbreeding or sampling related individuals. These eleven microsatellite markers are considered useful for population genetic studies and conservation decision-making for this medicinal plant.

## Figures and Tables

**Table 1:** Eleven microsatellite loci isolated for *D. huoshanense* collected from Huoshan County town: locus name, repeat motif, primer sequences, annealing temperature (*T*_a_), allele size range, number of alleles (*N*_a_), observed (*H*_o_) and expected (*H*_e_) heterozygosities and GenBank accession number.

Locus	Repeat motif	Primer sequence (5′-3′)	*T*_a_ (°C)	Allele size range (bp)	*N*_a_	*H*_o_	*H*_e_	GenBank Accession No.
Hs39 *	(TC)_26_	F: CACCGCTTGTCCATCAAACTTC R: CGCAATGGTTGAGCGTGTAAAT	61	313–357	4	0.227	0.582	JQ400190
Hs40	(TC)_20_	F: AAGATTGAGGCGATGTTGT R: ACTTGCTGGAATTTGTGGT	61	285–323	3	0.636	0.595	JQ400191
Hs41	(AG)_26_	F: CAGGCGGCAGAGTTACAG R: GCCAAAGCAAAACAAAGTAGAG	55	262–296	3	0.500	0.621	JQ400192
Hs42	(GA)_22_	F: TCCATCTATCCATCAACTAAGC R: GACGAGTATGTTCAGCAGATTG	61	340–360	2	0.364	0.488	JQ400193
Hs44	(CT)_16_	F: ATGGCATAAAACCTCCCCG R: GAGCGGACAATCGCATCTAAGT	55	215–287	4	0.818	0.757	JQ400194
Hs47	(TG)_10_(AG)_9_	F: TGAAATAGCCATCCTGAAGAG R: GGAGAAGATTATTGGGTTTGAGT	59	258–284	3	0.273	0.317	JQ400195
Hs48	(TG)_39_(AG)_30_(TG)_10_	F: AGGGAAAGGCCGTAATGATGAT R: AGAGTTGTAATGTTGGCTCCGCTA	61	294–382	5	0.636	0.719	JQ400196
Hs50 *	(TC)_28_	F: CCAAGCAAACCTCAAGAACTC R: CGAAAGGACCGACCTAATACT	52	283–305	3	0.455	0.671	JQ400197
Hs54	(TC)_18_C_3_(TC)_17_	F: CAGCCAACAGCCACTCTTCTTC R: CGCCGTCAAATAGTTTATCGTAG	59	227–293	3	0.500	0.515	JQ400198
Hs56	(AG)_6_A_3_(GA)_16_	F: GCAGAAGCAAGAATAAAAGTCG R: ATAAGATTAGATGAGCCCCAGA	59	212–240	2	0.545	0.495	JQ400199
Hs57	(TC)_22_	F: CCTTGAATTTGCATGGAGTT R: TTTTGGGGCATTTTGCTGTT	52	280–290	2	0.682	0.502	JQ400200

* indicates significant deviation from Hardy-Weinberg equilibrium (*p* < 0.05).

**Table 2:** Cross-species amplification of eleven microsatellite loci in five *Dendrobium* species.

Locus	*D. aduncum*	*D. henanense*	*D. moniliforme*	*D. nobile*	*D. officinale*
Hs39	226–279	190–279	310–369	238–268	190–226
Hs40	230–287	–	287–341	189–230	230
Hs41	322–380	323–395	359–395	322–380	323–359
Hs42	235–265	–	340–361	–	–
Hs44	279–316	279–320	300	268	268–314
Hs47	235–257	244–258	258–284	258	240–290
Hs48	310–380	240–290	300–375	240	286
Hs50	–	216–243	293	220–243	258–280
Hs54	193–211	220	230–301	193–230	–
Hs56	211–249	240–285	211–240	211–240	220–240
Hs57	225	210–225	280–300	210–280	230–268

The meaning of the provided values is the size range of PCR products and minus sign (–) denotes no visible PCR product.
